# Mismatch between student and tutor evaluation of training needs: a study of traumatology rotations

**DOI:** 10.1186/s13104-018-3925-1

**Published:** 2018-11-21

**Authors:** Fernando Santonja-Medina, Mari Paz García-Sanz, Sara Santonja-Renedo, Joaquín García-Estañ

**Affiliations:** 1 Departamento de Traumatología, Hospital Clínico Universitario Virgen de la Arrixaca, Instituto Murciano de Investigación Biomédica, Centro de Estudios en Educación Médica, Facultad de Medicina, Universidad de Murcia, Murcia, Spain; 2 Departamento de Educación, Facultad de Educación, Universidad de Murcia, Murcia, Spain; 3 Centro de Estudios en Educación Médica, Facultad de Medicina, Universidad de Murcia, Murcia, Spain; 4 Departamento de Fisiología, Instituto Murciano de Investigación Biomédica, Centro de Estudios en Educación Médica, Facultad de Medicina, Universidad de Murcia, Campus de El Palmar, 30120 Murcia, Spain

**Keywords:** e-Portfolio, Clinical skills, Competences, Medicine

## Abstract

**Objective:**

An e-portfolio was used to determine the optimal number of times students need to repeat a procedure before they are fully capable of performing it without supervision. The results were compared with the actual number of repetitions performed during the internship period. We also asked these students and their teachers about the optimal number of times each skill should be repeated before it could be considered fully acquired. The questionnaire was answered by 98.6% of the students and 70.3% of their teachers.

**Results:**

Both students and teachers agreed on a similar optimal value for 16 out of the 21 clinical procedures selected; in the remaining 5, teachers thought that students needed to repeat the procedure more times than the number stated by students. When these optimal values were compared with the actual values recorded in the portfolio during the internships, it was found that about half of all clinical procedures were carried out fewer times than expected, thus providing important feedback about the rotation-based training process. Quantitative information collected in the portfolios revealed a moderate mismatch between students’ and teachers’ perceptions of training needs.

## Introduction

The adoption of a system based on the acquisition of clinical competences [1] and the adoption of assessment instruments such as OSCEs in medical schools [2] is slowly changing the way medical teaching is carried out. Clinical competences relative to clinical skills and procedures require different teaching and learning mechanisms as well as, more importantly, different methods of ascertaining whether or not they have been fully acquired. Learning these clinical skills requires time, and procedures need to be repeated more than once before teachers can decide whether or not students are capable of performing them autonomously, or in other words, whether the required learning outcome has been achieved [3, 4]. However, determining the optimal number of times that students need to repeat a procedure in order to become competent enough to perform it independently remains a challenge in undergraduate medical education.

We have presented evidence that a reflexive portfolio is an important instrument for analyzing the acquisition of traumatology competences by medical students [5]. In this article, we describe the measures implemented to determine the number of times each student performs each procedure and compared it to the figures provided by both these same students and their teachers in the survey.

## Main text

### Methods

We used an updated version of our portfolio, described previously [5], to assess competences and learning outcomes assigned to Traumatology within the curriculum of the undergraduate medical degree run by the University of Murcia. This course is now in the second semester of the 4th year of a 6 year curriculum, and it has 6 ECTS (european credits), for a total of 60 presential hours (theory, seminars, skills lab and hospital internship) and 90 of student workload. Basically, the portfolio is now an electronic instrument (e-portfolio), divided into two different sections. The first is a qualitative and descriptive part in which students describe the activity carried out during their internship. The second section is a new quantitative part, in which they indicate the number of times the different learning tasks are repeated. There are 21 different learning tasks, all linked to the 5 competences that are evaluated using the e-portfolio. Students complete the e-portfolio during their Traumatology internship, which consists of a mandatory 10-day rotation (4 days in the outpatient clinic, 4 days in the operating room and 2 days in the emergency room). Students must complete this rotation in order to pass the subject, which accounts for 10% of their final grade. A total of 215 students were enrolled on the course and all of them had both personal and private access to the e-portfolio, which is located in a server at the University of Murcia (http://practicum-med.inf.um.es/portfolio/). Towards the end of the academic year, once the clinical skills exam had been completed, we administered a questionnaire to all participating students, and also to their hospital tutors (n = 74, all of them certified Specialists in traumatology), asking them how many times they believed each procedure should be repeated in order to acquire the corresponding skill or learning outcome. This questionnaire was made specially for the study and its reliability was very high (*Cronbach’s α of* 0.953 and 0.933, for students and teachers, respectively). Finally, we have also evaluated the performance of our students in the course assessment (multiple-choice test questions, skills examination, and image quiz). The data were analyzed using the Statistical Package for the Social Sciences (SPSS), version 19. Means and standard deviations were obtained to describe the results and the statistical significance between means was obtained with an ANOVA. A p value below 0.05 was considered to indicate statistical significance.

### Results

The questionnaire was completed by 212 of the 215 students enrolled (98.6%). The mean age of the students was 21.3 ± 0.9 years and they were 119 female and 93 male. The questionnaire was also administered to faculty members teaching on the internship, which was completed by 52 out of 74 (70.3%). Their age was 51.3 ± 6.2 years and they were 9 female and 43 male doctors. Table 1 shows the comparison between the estimated number of times that students and their teachers said they thought it was necessary to repeat a procedure in order to consider it effectively acquired. In most cases (16 out of 21), the values indicated by students and teachers were very similar. In 5 cases, however, the number given by teachers was statistically higher than that indicated by students. Regarding the performance of the students (Table 1) in comparison with these optimal numbers, we found that almost half of the procedures (12/21 according to students and 11/21 according to teachers), were repeated fewer times than the optimal value. The rest of the procedures were performed more often and exceeded this optimal value. In the assessment of the course, the mean grades obtained, grouped by competences, were competence 1: 6.72/10; competence 2: 9.54/10; competence 3: 6.75/10; competences 4 & 5: 8.29/10.

**Table 1:** Optimal number of times that students and teachers said they thought it was necessary to repeat a procedure in order to achieve the learning outcome (* p < 0.05 between students and teachers)

Competences	Learning outcomes	Optimal values		Compared to optimal value of		
		Students	Teachers	Observed value	Students	Teachers
1. Recognize injuries, assessment and consequences	Assessment of wound severity	6.39 ± 4.41	8.69 ± 6.89*	1.72 ± 2.12	Lower*	*Lower* ^+^
	Fracture diagnosis	7.63 ± 5.74	11.19 ± 9.49*	5.55 ± 3.64	Lower*	*Lower* ^+^
	Diagnosis of ligament injuries	8.32 ± 6.70	10.15 ± 6.35	1.72 ± 2.12	Lower*	*Lower* ^+^
	Diagnosis of muscle lesions	8.58 ± 6.41	7.85 ± 5.53	0.51 ± 1.06	Lower*	*Lower* ^+^
	Diagnosis of tendinopathy	8.66 ± 5.86	9.60 ± 5.66	0.85 ± 1.37	Lower*	*Lower* ^+^
2. Identify lesions during physical examination	Assessment of omalgia	7.27 ± 5.38	8.67 ± 6.08	7.59 ± 7.16	NS	NS
	Assessment of gonalgia/coxalgia	7.39 ± 6.03	8.96 ± 6.31	21.95 ± 13.57	Higher*	Higher ^+^
	Assessment of cervical/back pain	7.45 ± 6.06	7.37 ± 5.45	10.32 ± 12.92	Higher*	Higher ^+^
	Assessment of lumbar pain	7.51 ± 6.13	7.90 ± 6.05	5.68 ± 7.81	Lower*	*Lower*
	Examination of ankle and foot	7.10 ± 4.99	7.92 ± 5.26	12.34 ± 9.49	Higher*	Higher ^+^
	Examination of elbow, wrist and hand	8.34 ± 5.73	9.85 ± 7.07	10.77 ± 9.72	Higher*	NS
	Examination of backbone: scoliosis/kifosis/lordosis	8.05 ± 5.35	9.75 ± 6.59	2.26 ± 3.14	Lower*	*Lower* ^+^
3. Recognize lesions using imaging techniques	Reading X-rays	8.83 ± 8.83	12.90 ± 15.67	60.45 ± 38.51	Higher*	Higher ^+^
	Reading magnetic resonance images	11.00 ± 11.00	16.73 ± 15.94*	22.23 ± 17.03	Higher*	Higher ^+^
	Reading computerized axial tomography images	10.17 ± 10.17	14.15 ± 10.99*	5.42 ± 7.69	Lower*	Higher ^+^
4. Orthopaedic treatment of lesions (non surgical, supervised)	Evaluation of immobilizations	7.96 ± 6.65	10.27 ± 8.96	5.66 ± 5.42	Lower*	*Lower* ^+^
	Healing of wounds	6.38 ± 8.36	8.65 ± 6.81*	14.39 ± 10.20	Higher*	Higher ^+^
	Performing articular infiltration	9.90 ± 7.61	9.02 ± 7.32	0.76 ± 1.47	Lower*	*Lower* ^+^
5. Observe and assist in surgical treatments	Assisting in the operating room	6.56 ± 5.89	6.94 ± 4.42	8.38 ± 3.52	Higher*	Higher ^+^
	Surgical washing	8.14 ± 7.42	8.67 ± 5.97	4.24 ± 3.12	Lower*	*Lower* ^+^
	Suture of wounds in the emergency unit	13.00 ± 12.52	18.10 ± 19.48	7.27 ± 3.47	Lower*	*Lower*

The observed value shows the actual number of times that the procedures are performed during the hospital internship. The last two columns compare the optimal value with the observed value, both for students and teachers. * p < 0.05 vs optimal mean of students; ^+^ p < 0.05 vs optimal mean of teachers; *NS* not significant

### Discussion

The e-portfolio used in this study enables an improved assessment of the activities carried out by medical students during internships. By including a quantitative element where they provide information regarding the number of times each task is performed, we can easily ascertain whether or not students have acquired the necessary competences and whether the 10 days for which the rotation lasts should be distributed differently among the outpatient clinic, emergency room and operating room, or whether other, alternative strategies are required.

First, we have determined the optimal number of times that each one of the proposed skills or procedures should be repeated. Interestingly, the study shows that both students and their teachers agreed on a similar optimal value for 16 out of the 21 clinical procedures selected; and only in 5 of them did teachers think the maneuver needed to be repeated more times than the number indicated by students, thus indicating a moderate mismatch between teachers’ and students’ perceptions of training needs. There are multiple possible reasons for this that are interesting to analyze. Regarding competence 1, the results indicate that the procedures were performed fewer times than the indicted optimal value. But, many of these concepts are taught also in the theoretical classes and seminars, and some conditions such as muscular or tendon injuries have a low frequency of presentation. The acquisition levels for both competences 2 and 3 were high, with some procedures being repeated many more times than the optimal values. Competence 4 has two learning outcomes with values well below the optimal values. These are procedures that students must learn to master, at least for the immobilizations and participation in arthrocentesis. Teachers should therefore be asked to encourage students’ more active participation in these maneuvers. However, they see many wounds and know how to manage them. Similarly, competence 5, which is related to the operating room, also has repetition values lower than the optimal ones. This should be a focus of our attention, and we should strive to help students increase the number of surgical washing and suturing procedures they carry out, although these tasks are also included in the general surgery rotation.

From the results obtained in this study, it can be concluded that the e-portfolio is a tool that provides information which would be difficult to obtain using more traditional assessment instruments [6]. However, it is not a substitute for those instruments, but rather complements their use [7], helping to improve the quality of the evaluation conducted of both the process itself and the institution in which it is implemented. As observed with the assessment grades, our students reasonably obtained all these minimum competences to pass the course.

## Limitations

The main limitation of our study is that the results obtained are based on student and tutor perceptions regarding the number of times an array of procedures need to be done in order to determine the competency while performing them. However, we think that this number is of interest since it is an indication of the level that students should reach in order to attain the competence. We are working towards the definition of this minimum number throughout consultation with other Medical Schools in Spain.
